# Caregiver‐reported dementia as a predictor of oral health among patients receiving home‐visit dental treatment: A retrospective cohort study

**DOI:** 10.1002/cre2.333

**Published:** 2020-10-21

**Authors:** Satoshi Yamaguchi, Yukari Horigome, Kosei Endo, Moritoshi Komagata, Shinya Komai, Kenichiro Komaki, Hideki Miyata, Kazuhiko Sugano, Setsuko Ito, Shiho Itabashi, Harumi Sato, Minako Okahashi, Sayaka Kishi, Rika Abe, Yoshinori Hattori

**Affiliations:** ^1^ Division of Aging and Geriatric Dentistry Tohoku University Graduate School of Dentistry Sendai Japan; ^2^ Geriatric and Prosthetic Dentistry Tohoku University Hospital Sendai Japan; ^3^ Sendai Dental Association Sendai Japan

**Keywords:** dementia, Japan, oral health, risk factors

## Abstract

**Objective:**

To assess caregiver‐reported dementia as a risk factor for retained roots, an indicator of poor oral hygiene, among patients receiving home‐visit dental treatment in Japan.

**Methods:**

The medical records of 231 dentate patients who received home‐visit dental treatment (covered by public medical insurance) for more than 2 years were retrospectively analyzed. The number of teeth and retained roots at the initial and final examinations were obtained from the dental charts, and the “change in the number of retained roots from initial to final examination” was determined. The presence or absence of caregiver‐reported dementia, diabetes, and osteoporosis, as well as the level of long‐term care needed, were used as indicators of general health condition at the initial interview. Multiple regression analyses were conducted in five models that tested the association of independent variables (age, gender, observation period, general health, presence or absence of caregiver‐reported dementia at the initial interview) with changes in the number of retained roots.

**Results:**

In all models, the presence of caregiver‐reported dementia at the initial interview was significantly associated with the change in the number of retained roots (*p* < .05). The adjusted coefficient of determination (*R* ^2^) of model 5, which included all the predetermined independent factors, was .168.

**Conclusions:**

Caregiver‐reported dementia may be a risk factor for an increase in the number of retained roots among patients who receive home‐visit dental treatment and may serve as an indicator of the need for regular and proactive oral hygiene management.

## 1 INTRODUCTION

The increasing number of older people with dementia is a common global and social issue and is expected to reach 131.5 million by 2050 (Alzheimer's Disease International (ADI), 2016). Dementia is a known risk factor for the deterioration of oral health (Delwel et al., 2017) and this may be partly attributed to a decreased utilization of dental care due to difficulties in visiting a dental office (Lee, Wu, & Plassman, 2015). It is important to evaluate the effectiveness of home‐visit dental treatment for patients with dementia, as home‐visit dental treatment may be effective in increasing the utilization of dental care for patients who have difficulties in accessing a dental office.

Japan has the most aged society in the world, and public medical insurance covers most of the costs of self‐requested home‐visit dental treatment. Hence, there are a very large number of home‐visit dental treatments for patients with dementia and others who have difficulty visiting a dental office. However, due to limited treatment time and medical resources, it is difficult for the dentist to confirm the presence of a physician's diagnosis of dementia or to perform a cognitive assessment during the home‐visit dental treatment. Often only caregiver‐reported information (proxy‐reported physician's diagnosis of dementia or the caregiver's subjective estimation of the patient's dementia) can be obtained by interviewing the patient's family or facility staff. Therefore, it is worthwhile to assess the impact of caregiver‐reported dementia on the subsequent oral health of patients, even if a physician's diagnosis of dementia or degree of dementia is unknown, to develop strategies for providing home‐visit dental treatment with limited medical resources. However, no studies have investigated the effect of caregiver‐reported dementia on oral health in actual cases of home‐visit dental treatment, although dementia has been shown to have a deleterious effect on oral health among older people who are nursing home residents (Adam & Preston, 2006). There remains a need for the determination of whether caregiver‐reported dementia is a risk factor for deteriorating oral hygiene, using data from actual home‐visit dental treatments. This would facilitate the assessment of high‐risk patient groups and help in the development of optimal oral care protocols.

There are issues that need to be considered in extracting and analyzing data from the medical records of actual home‐visit dental treatments. Given that the majority of the patient complaints pertain to denture problems and that the dental care providers often have limited time for completing patient charts, data on periodontitis and dental caries may be inaccurate. Therefore, we considered that the number of retained roots, which is relatively reliable even when assessed through limited clinical examinations, was an appropriate and objective indicator of oral health status. Indeed, the number of retained roots has been utilized as an oral health indicator in a previous study conducted among patients with dementia (Chalmers, Carter, & Spencer, 2003; Chen, Clark, & Naorungroj, 2013).

Thus, the objective of this study was to assess caregiver‐reported dementia as a risk factor for retained roots among patients receiving home‐visit dental treatment in Japan. Confirmation of this hypothesis would suggest that caregiver‐reported dementia is a major risk factor for the deterioration of oral health, even in cases without a formal medical diagnosis of dementia.

## 2 STUDY POPULATION AND METHODOLOGY

### 2.1 Study population

This study was conducted as part of an epidemiological study in adult patients, who received home‐visit dental treatment (either at home, at a hospital, or at a nursing home in Sendai, Japan) by dental care providers from the Sendai Dental Association and the Division of Aging and Geriatric Dentistry, Tohoku University Graduate School of Dentistry. Altogether, 4,486 patients underwent their first dental examination between 2005 and 2013. Among these patients, 231 had dental records over a consecutive 2‐year period. Following the exclusion of 30 edentulous patients, data were extracted from the dental records of the remaining 201 patients. This research was conducted with the approval of the Research Ethics Committee of the Tohoku University Graduate School of Dentistry (receipt number: March 2, 2016). Informed consent in the form of the “opt‐out method” was made available on the website.

### 2.2 Data acquisition

Medical records were written on paper for home‐visit dental treatment only, not for research purposes. Home‐visit dental treatments were conducted on an irregular basis at the request of patients or caregivers. Information about the general condition of the patient was obtained by a questionnaire completed by the caregiver at the time of application for the home‐visit dental treatment and by questioning the caregiver and patient at the first visit of the dental treatment. For systemic diseases, including dementia, only the presence or absence of disease was asked; the presence or absence of a definitive diagnosis and the level and duration of the disease were not recorded. Intraoral condition was recorded by visual inspection, at the initial visit as well as at irregular dental visits as needed.

Data from the medical records were entered into a database (FileMaker Pro 14 Advanced), with all personal identifiers removed. The database included each patient's sociodemographic information and medical history (gender, age, date of first examination, presence or absence of a family doctor, degree of care required, chief dental complaint, reason for inability to visit a hospital, any systemic illness), as well as the intraoral condition (reasons for dental treatment during home visits, dental chart, use of a removable denture). As the dental charts had been completed at the initial examination and were updated at irregular home‐visit dental treatments, it was possible to determine changes in the intraoral condition (the number of teeth, the number of retained roots, the number of carious teeth, and the number of missing teeth).

### 2.3 Data processing

In this study, (a) the number of teeth including retained roots at the initial examination, (b) the number of retained roots at the initial examination, and (c) the changes in the number of retained roots from the initial to the final examination (the number of retained roots at the final examination minus the number of retained roots at the initial examination) were calculated from the dental charts. Items (a) and (b) represented the state of the oral cavity at the initial examination, while item (c) represented the change in the state of the oral cavity. As items (a) and (b) did not have a normal distribution, item (a) was categorized into tertiles (1–8 teeth: *n* = 63, 9–9 teeth: *n =* 67, 20–32 teeth: *n =* 71) and item (b) was categorized into “presence or absence of retained roots at initial examination” using the median value (with no retained roots: *n =* 104, with retained roots: *n =* 97).

Only the presence or absence of primary systemic illnesses was documented in the medical records, and there was no distinction between the proxy‐reported physician's diagnosis (as obtained by interviewing the patient's family) and the caregiver's subjective determination. Although few systemic diseases have been shown to affect oral health, diabetes and osteoporosis are proven risk factors for periodontal disease(Genco & Borgnakke, 2013; Kuo, Polson, & Kang, 2008) and may affect changes in the number of retained roots; therefore, we considered these diseases as covariates. As our home‐visit dental treatments covered not only personal residences but also nursing homes and hospital wards, varying prevalences of dementia, diabetes, and osteoporosis were recorded. However, there was no information in the database on disease severity. Therefore, in addition to “dementia,” both “diabetes” and “osteoporosis” were categorized into two groups (presence or absence of caregiver‐reported dementia, presence or absence of caregiver‐reported diabetes, and presence or absence of caregiver‐reported osteoporosis), based on the presence or absence of caregiver‐reported systemic illness at the initial examination. These represented the patient's general condition at the initial examination. The degree of care required at the initial examination, which may be related to the initial state of both dementia and oral hygiene, was also used as a covariate. This was categorized into two groups: “support level 1–2 or care level 1–2” (*n =* 92) and “care level 3–5” (*n =* 109), as certified by the Japanese public long‐term care insurance system. This system has seven eligibility levels, including support levels 1–2 and care levels 1–5 (Kamiya, Adachi, Sasou, Suzuki, & Yamada, 2017). This study used medical record data from home‐visit dental treatment conducted at the request of patients and caregivers, so the duration of the observation period varied greatly from patient to patient. The number of days from the first visit to the last examination (days of observation period) was calculated and used as a variable to adjust for the observation period.

### 2.4 Statistical analysis

The *χ*² test and *t* test were used to analyze differences among the patients in terms of sociodemographic and clinical variables, including the presence or absence of caregiver‐reported dementia, at the initial examination. Multiple regression analyses were performed in the following five models, with changes in the number of retained roots as the dependent variable and the presence or absence of caregiver‐reported dementia at the initial examination as the independent variable: Model 1 (unadjusted), Model 2 (adjusted for “age” and “gender”), Model 3 (in addition to model 2, adjusted for the “presence or absence of caregiver‐reported diabetes,” “presence or absence of caregiver‐reported osteoporosis,” “number of teeth including retained roots at initial examination,” and “presence or absence of retained roots at initial examination”), Model 4 (in addition to Model 3, adjusted for “degree of care required”), and Model 5 (in addition to Model 4, adjusted for “days of observation period”).

Statistical analyses were performed using SPSS Statistics 22.0 (New York: IBM Corp, 2013). *p* values < .05 were considered significant.

## 3 RESULTS

Table 1 shows the basic sociodemographic and clinical characteristics of the 201 patients (average age, 76.1 ± 12.3 years; age range, 23–97 years; 65.2% women) at the initial examination. The proportions of patients with dental caries and missing teeth were 48.4% and 98.5%, respectively. With regard to the “reason for the patients' inability to visit hospitals,” walking difficulties were the most frequently reported cause (79.6%). Dementia was cited as the cause among 40.3% of the patients, while less than one‐fifth (14.9%) reported being bedridden (multiple answers were possible). Denture adjustment was the most frequent reason (36.8%) for home visits for dental treatment, and this increased to 76.1% when combined with the need for caries and periodontal treatment.

**TABLE 1:** Basic characteristics at initial examination

Number of patients, *n* (%)	201 (100)
Age, mean ± SD (range)	76.1 ± 12.3 (23–79)
Gender, *n* (%)	
Male	70 (34.8)
Female	131 (65.2)
Number of teeth, *M* ± *SD*	13.3 ± 9.1
Number of teeth including retained roots, *M* ± *SD*	14.9 ± 9.1
Number of retained roots, *M* ± *SD*	1.5 ± 2.5
Number of carious teeth, *M* ± *SD*	1.6 ± 2.6
Number of missing teeth, *M* ± *SD*	5.6 ± 3.9
Proportion of patients with dental caries, *n* (%)	98 (48.8)
Proportion of patients with missing teeth, *n* (%)	198 (98.5)
Reasons for inability to visit hospitals, *n* (%)^a^	
Walking difficulties	160 (79.6)
Dementia	81 (40.3)
Wheelchair	69 (34.3)
Bedridden	30 (14.9)
Other	80 (39.8)
Reasons for dental treatment during home visits, *n* (%)	
Denture adjustment	74 (36.8)
Caries treatment	42 (20.9)
Periodontal treatment	37 (18.4)
Detachment of prosthesis	20 (10.0)
New denture made	12 (6.0)
Oral care	8 (4.0)
Tooth extraction	6 (3.0)
Other	2 (1.0)

Abbreviations: *n*, number; *SD*, standard deviation.

^a^ Multiple answers were possible.

Table 2 shows the basic characteristics of the patients, divided by the presence or absence of caregiver‐reported dementia, at the initial examination. Eighty‐seven (43.3% of the total) patients had caregiver‐reported dementia, and they were significantly older than those without a description of dementia. A significantly higher proportion of patients (17.2%) in the group with caregiver‐reported dementia had osteoporosis compared to those in the group without caregiver‐reported dementia (5.3%; *p* = .006). Likewise, the proportion of patients who had 20 or more teeth (including retained roots) was significantly lower in the group with caregiver‐reported dementia (21.8%), compared to that in the group without caregiver‐reported dementia (45.6%; *p* = .002). No significant differences in other variables were found between these two groups at the initial examination.

**TABLE 2:** Basic characteristics by presence or absence of caregiver‐reported dementia at initial examination

		All (*n* = 201)	Caregiver‐reported dementia		*p* value^a^
			Presence (*n* = 87)	Absence (*n* = 114)	
Age, *M* ± *SD*		76.1 ± 12.3	81.7 ± 8.1	71.9 ± 13.3	<.001
Gender, *n* (%)					.113
Male		70 (34.8)	25 (35.7)	45 (39.5)	
Female		131 (65.2)	62 (71.3)	69 (60.5)	
Caregiver‐reported diabetes, *n* (%)					.995
Presence		30 (14.9)	13 (14.9)	17 (14.9)	
Absence		171 (85.1)	74 (85.1)	97 (85.1)	
Caregiver‐reported osteoporosis, *n* (%)					.006
Presence		21 (10.4)	15 (17.2)	6 (5.3)	
Absence		180 (89.6)	72 (82.8)	108 (94.7)	
Number of teeth including retained roots, *n* (%)					.002
1–8 teeth		63 (31.3)	31 (35.6)	32 (28.1)	
9–19 teeth		67 (33.3)	37 (42.5)	30 (26.3)	
20–32 teeth		71 (35.3)	19 (21.8)	52 (45.6)	
Number of retained roots, *n* (%)					.772
Presence		97 (48.3)	43 (49.4)	54 (47.4)	
Absence		104 (51.7)	44 (50.6)	60 (52.6)	
Degree of care required, *n* (%)					.096
Care level 2 or less		92 (45.8)	34 (39.1)	58 (50.9)	
Care level 3 or more		109 (54.2)	53 (60.9)	56 (49.1)	
Days of observation period, *M* ± *SD*		1,463.1 ± 423.7	1,458.4 ± 415.8	1,466.6 ± 431.5	.892

Abbreviations: *n*, number; SD, standard deviation.

^a^ Student's *t* test (“Age” and “Observation period”) and *χ*² (others).

The results of the multiple regression analyses in each model are shown in Table 3. Changes in the number of retained roots, which was the dependent variable, ranged from −10 (patient with the largest reduction in the number of retained roots) to +13 (patient with the greatest increase in the number of retained roots). In all models, the presence or absence of caregiver‐reported dementia at the initial examination was significantly associated with the change in the number of retained roots (*p* < .05). The adjusted coefficient of determination (*R* ^2^) of model 5 was .168.

**TABLE 3:** Multiple regression analysis with the change in the number of retained roots as the dependent variable

	Model 1		Model 2		Model 3		Model 4		Model 5	
	*β*	*p*	*β*	*p*	*β*	*p*	*β*	*p*	*β*	*p*
Presence or absence of caregiver‐reported dementia	.152	.031	.165	.033	.183	.011	.174	.015	.174	.015
Age			−.031	.696	.102	.208	.082	.311	.081	.316
Gender			−.005	.950	.054	.442	.079	.272	.074	.300
Presence or absence of caregiver‐reported diabetes					.042	.531	.021	.748	.016	.806
Presence or absence of caregiver‐reported osteoporosis					−.128	.065	−.129	.061	−.131	.059
Number of teeth including retained roots at initial examination					.238	.002	.240	.002	.240	.002
Presence or absence of retained roots at initial examination					−.344	.000	−.344	.000	−.345	.000
Degree of care required							.123	.068	.136	.048
Days of observation period									.066	.317

Abbreviation: *β*, standardized partial regression coefficient.

## 4 DISCUSSION

In this study, data extracted from the medical records of patients receiving home‐visit dental treatment, covered by public medical insurance, were analyzed longitudinally. It was found that caregiver‐reported dementia, in the absence of a formal medical diagnosis, may be a risk factor for an increased number of retained roots. The increase in the number of retained roots is considered to reflect a deterioration of oral hygiene.

The caries prevalence in this study was higher than that reported by the National Survey of Dental Diseases conducted in Japan in 2011, which found that 39.3% of elderly individuals aged between 75 and 84 years had untreated dental caries (Table 1) (Ministry of Health, Labour and Welfare, Japan, 2011). While this may reflect the poorer oral health of the patients who required home visits for dental treatment in this study, we speculate that the reliability of caries diagnosis may have been lower, given that the examinations were conducted in a field setting and not under controlled clinical conditions. It is considered that the caries prevalence could have been underestimated, as the examinations performed during the home visits were largely exploratory in nature, and radiographs were not used to aid in the diagnosis of interproximal caries. On the contrary, we considered the reliability of the number of retained roots to be high because the possibility that missing teeth and retained roots are overlooked is low even when the dental treatment during home visits is only exploratory. The results in Table 1 show that few patients with severe dementia were bedridden, and that many patients required general dental management such as denture adjustment and restorative treatment for caries. The results in Table 2 show that the presence or absence of dementia might be related not only to age but also to the presence or absence of osteoporosis. However, it should be taken into account that the age difference between the two groups was very large.

In this study, the number of retained roots was used as an indicator of oral hygiene. Root caries and retained roots are characteristic findings among older individuals (Delwel et al., 2017; Petersen & Yamamoto, 2005), particularly in patients requiring home‐visit dental treatment. The use of the number of retained roots as an indicator of the quality of the oral environment has already been established in prior studies (Chalmers et al., 2003; Chen et al., 2013). Furthermore, as documentation of each patient's oral hygiene status and periodontal condition was not available in the dental records, it was reasonable to use the change in the number of retained roots as an indirect indicator of the level of oral hygiene.

In this study, multiple regression analyses were performed using five models with different independent variables. As a strong relationship was expected between the “degree of care required” and “dementia,” models containing the former variable were individually created for the other independent variables (Model 4). As the observation periods could not be standardized, the number of days elapsed from the initial examination to final examination was defined as the “observation period” (a continuous variable), and this was included as an independent variable in Model 5. The “presence or absence of caregiver‐reported dementia” was found to be a significant factor affecting the number of retained roots in all models. Models 1, 2, and 3 showed that the “presence of caregiver‐reported dementia” at the initial examination was associated with an “increase in the number of retained roots,” and that this was independent of “age,” “gender,” “presence or absence of caregiver‐reported diabetes at initial examination,” “presence or absence of caregiver‐reported osteoporosis at initial examination,” and “current number of teeth including retained roots at initial examination.” Models 4 and 5 confirmed that the influence of “degree of care required” and “observation period” was not substantial. Previous studies have reported an increase in the number of retained roots in community‐dwelling elderly patients with dementia (Chalmers et al., 2003), which support the results of this present study. In model 5, the standardized partial regression coefficients for “presence or absence of caregiver‐reported diabetes,” “presence or absence of caregiver‐reported osteoporosis,” and “presence or absence of caregiver‐reported dementia” were 0.016, −0.131, and 0.174, respectively. The results of our study confirm that dementia is a risk factor for poor oral hygiene, unlike diabetes and osteoporosis, which have been reported to directly affect periodontal health (Genco & Borgnakke, 2013; Kuo et al., 2008).

Patients with caregiver‐reported dementia were found at a higher risk for deterioration of oral hygiene, despite receiving home‐visit dental treatment. This suggests that the frequency and method of home‐visit dental treatment, as currently provided under the medical insurance system in Japan, are insufficient to maintain an optimal level of oral health among patients with dementia. The reliability of caregiver‐reported dementia in this study is unclear because the physician's diagnosis of dementia was not recorded. However, as memory problems are most often the first symptoms of dementia in Alzheimer's disease, caregivers have an integral role in the early detection of this condition (Montgomery, Goren, Kahle‐Wrobleski, Nakamura, & Ueda, 2018). There is often no time to confirm the presence of a physician's diagnosis of dementia at home‐visit dental treatments under the Japanese public medical insurance system. However, if caregiver‐reported dementia is confirmed to be a risk factor for oral health, it would be beneficial for dental providers to use caregiver‐reported information for the identification of patients who exhibit early signs and symptoms, even in the absence of a formal medical diagnosis, and provide appropriate preventive dental management. While the results of this study provide an initial evidence for the analysis of the efficacy of periodic home‐visit dental treatment, additional longitudinal studies are required to help in the further development and improvement of measures designed to maintain the oral health of patients with dementia.

Some limitations of this study should be acknowledged. The data used in this study were not collected specifically for this analysis and were extracted from the medical records obtained during home‐visit dental treatment. Therefore, the questions asked during the interview and the method of examination will vary depending on the dentist assigned to the case. The definitive diagnosis of dementia and the duration and level of the dementia are unknown, and the relationship between caregiver‐reported dementia, the physician's diagnosis of dementia, and the duration and level of the dementia needs to be investigated in future studies. In the statistical analysis, the different observation periods for each patient were not strictly considered. Additionally, while dental treatment and oral care were performed within the observation period in most cases, the number and type of treatments varied widely and were not accounted for in the statistical analysis. Furthermore, as all home‐visit dental treatments included in this study were covered by the public medical insurance in Japan, the results of this study may not be generalizable to all home‐visit dental treatments.

In conclusion, the results of this study suggest that caregiver‐reported dementia, in the absence of a formal and definitive diagnosis, may be a predictor for an increased number of retained roots in patients receiving home‐visit dental treatment covered by medical insurance in Japan. This could reflect the progression of severe cervical caries due to the deterioration of oral hygiene and could indicate the need for improved preventive dental management in this patient group.

## CLINICAL RELEVANCE

### Scientific rationale for the study

Patients with caregiver‐reported dementia, in the absence of a definitive diagnosis, may be at risk for deterioration of oral hygiene while receiving concurrent home‐visit dental treatment.

### Principal findings

Dementia, as reported by a caregiver at the first home‐visit dental consultation, was found to be a risk factor for the subsequent increase in the number of retained roots, which reflected a deterioration in the level of oral hygiene.

### Practical implications

Regular and proactive oral hygiene management should be implemented for patients with caregiver‐reported dementia, even in the absence of a definitive diagnosis.

## CONFLICT OF INTEREST

The authors declare no conflict of interest.

## AUTHOR CONTRIBUTIONS

Satoshi Yamaguchi the corresponding author, designed the study, performed statistical analysis of the data, and drafted the paper; Yukari Horigome and Kosei Endo constructed a database from the medical records; Moritoshi Komagata, Shinya Komai, Kenichiro Komaki, Hideki Miyata, Kazuhiko Sugano, Setsuko Ito, and Shiho Itabashi created and managed the medical records; Harumi Sato, Minako Okahashi, Sayaka Kishi, and Rika Abe assisted in the creation and management of the medical records; Yoshinori Hattori supervised the project and advised on the interpretation of data.

## Data Availability

The data that support the findings of this study are available on request from the corresponding author. The data are not publicly available due to privacy or ethical restrictions.

## References

[cre2333-bib-0001] Adam, H. , & Preston, A. J. (2006). The oral health of individuals with dementia in nursing homes. Gerodontology, 23(2), 99–105. 16677183 10.1111/j.1741-2358.2006.00118.x

[cre2333-bib-0002] Alzheimer's Disease International (ADI) . (2016). World Alzheimer Report 2016: improving healthcare for people living with dementia: coverage, quality and costs now and in the future. Retrieved January 20, 2020 from https://www.alz.co.uk/research/WorldAlzheimerReport2016.pdf

[cre2333-bib-0003] Chalmers, J. M. , Carter, K. D. , & Spencer, A. J. (2003). Oral diseases and conditions in community‐living older adults with and without dementia. Special Care in Dentistry, 23(1), 7–17. 12887148 10.1111/j.1754-4505.2003.tb00283.x

[cre2333-bib-0004] Chen, X. , Clark, J. J. , & Naorungroj, S. (2013). Oral health in nursing home residents with different cognitive statuses. Gerodontology, 30(1), 49–60. 22364512 10.1111/j.1741-2358.2012.00644.x

[cre2333-bib-0005] Delwel, S. , Binnekade, T. T. , Perez, R. S. , Hertogh, C. M. , Scherder, E. J. , & Lobbezoo, F. (2017). Oral health and orofacial pain in older people with dementia: A systematic review with focus on dental hard tissues. Clinical Oral Investigations, 21(1), 17–32. 27631597 10.1007/s00784-016-1934-9 PMC5203832

[cre2333-bib-0006] Genco, R. J. , & Borgnakke, W. S. (2013). Risk factors for periodontal disease. Periodontology 2000, 62(1), 59–94. 23574464 10.1111/j.1600-0757.2012.00457.x

[cre2333-bib-0007] Kamiya, K. , Adachi, T. , Sasou, K. , Suzuki, T. , & Yamada, S. (2017). Risk factors for disability progression among Japanese long‐term care service users: A 3‐year prospective cohort study. Geriatrics & Gerontology International, 17(4), 568–574. 27098728 10.1111/ggi.12756

[cre2333-bib-0008] Kuo, L. C. , Polson, A. M. , & Kang, T. (2008). Associations between periodontal diseases and systemic diseases: A review of the inter‐relationships and interactions with diabetes, respiratory diseases, cardiovascular diseases and osteoporosis. Public Health, 122(4), 417–433. 18028967 10.1016/j.puhe.2007.07.004

[cre2333-bib-0009] Lee, K. H. , Wu, B. , & Plassman, B. L. (2015). Dental care utilization among older adults with cognitive impairment in the USA. Geriatrics & Gerontology International, 15(3), 255–260. 24612371 10.1111/ggi.12264 PMC4145033

[cre2333-bib-0010] Ministry of Health, Labour and Welfare, Japan . (2011). Statistical tables of the survey of dental diseases (2011) Part 1. Retrieved January 20, 2020 from http://www.mhlw.go.jp/toukei/list/dl/62-17c23-1.pdf

[cre2333-bib-0011] Montgomery, W. , Goren, A. , Kahle‐Wrobleski, K. , Nakamura, T. , & Ueda, K. (2018). Detection, diagnosis, and treatment of Alzheimer's disease dementia stratified by severity as reported by caregivers in Japan. Neuropsychiatric Disease and Treatment, 14, 1843–1854. 30038495 10.2147/NDT.S160591 PMC6052934

[cre2333-bib-0012] Petersen, P. E. , & Yamamoto, T. (2005). Improving the oral health of older people: The approach of the WHO global Oral health Programme. Community Dentistry and Oral Epidemiology, 33(2), 81–92. 15725170 10.1111/j.1600-0528.2004.00219.x

